# AI Mimicking and Interpreting Humans: Legal and Ethical Reflections

**DOI:** 10.1007/s11673-025-10424-9

**Published:** 2025-06-12

**Authors:** J. M. Paterson

**Affiliations:** University of Melbourne, 185 Pelham Street, Carlton, Victoria 3053 Australia

**Keywords:** Artificial intelligence, Ethics, Law, AI companions, AI influencers, Mental health therapy chatbot, Emotion detection AI

## Abstract

The increasing prevalence of AI in all facets of human lives raises profound questions of ethics, policy, and law. Interactions with AI in situations that traditionally involve humans demonstrate the growing sophistication and adaptivity of the technology. For this very reason, we may demand some basic rules of engagement from these interactions—AI should not deceive humans into believing it is human or that it has human-like capacities and should be transparent about its artificial status. Law increasingly makes these demands. We may further question as a matter of practical ethics, if not law, whether even “well-trained” AI should be used at all in intimate or personal interactions with humans. This essay seeks to explore these issues by reference to a series of examples in which AI seeks to mimic or interpret humans: AI influencers on social media, AI companions, AI mental health therapy chatbots, and AI emotion detection tools.

## Introduction

Artificial intelligence (AI) and related data-driven technologies are becoming more prevalent and sophisticated across both public and private sectors. This development undoubtedly presents opportunities to benefit society, such as by providing new insights from data and performing mundane tasks more efficiently. The growing use of AI also raises risks of harm, including relating to bias, privacy, error/hallucination, job displacement, environmental impact, and malicious third-party use (Slattery et al. 2024). As a result, many countries are considering or have enacted specific laws to respond to concerns about the risk of harm arising from uses of AI (Yang and Bernot 2023). One concern is the risk of AI informing erroneous decisions, impacting heavily on individuals already experiencing vulnerability and threatening rule of law values (Royal Commission into the Robodebt Scheme 2023, 488; Ng 2021, 916–917). Another concern arises from malicious uses of generative AI to create deep fakes for fraud, abuse, and misinformation, harming individuals (Paterson 2024) and democratic values (Smith and Masted 2020).

This essay considers a more subtle set of concerns about the risks of AI, which arise when this technology is used in personal or intimate human interactions to provide advice, companionship, or support. Certainly, AI may offer opportunities for extending previously unavailable services or companionship to people. However, for the very reason that it is capable of doing this, we might demand some basic rules of engagement. These rules might simply be that AI should not mislead humans about its identity or capacity in any interaction with them. As is increasingly common in AI governance and legal regimes, this would require transparency about the uses of AI that might not be self-evident. Some AI-human interactions are founded on a pretence of understanding or empathy on the part of the AI. In these cases, the law might require not only disclosure of the artificial nature of the agent on one side of the transaction but also the limitations on its capacity to understand or feel. It might even be proposed that AI should not be used in deeply personal or intimate interactions with humans.

How far the rules should go and whether they should lead to legal restrictions or even bans in some instances are not questions the law can answer alone. Society needs to consider whether as a matter of ethics or policy there are some kinds of AI–human interactions that should not be encouraged at all, even if operating without the concerns of bias or hallucination that plague our current AI models.

This essay seeks to explore these issues by reference to a series of examples: AI influencers on social media, AI virtual companions, AI mental health therapy chatbots, and AI emotion detection tools. Part II considers why we might be concerned about the deceptive or misleading potential of AI-to-human interactions. Part III considers examples of AI mimicking humans: AI influencers on social media andAI companions. Part IV considers a different variant of AI-human interactions, arising where AI is presented as having the capacity to understand human behaviour or emotion: AI chatbots offering mental health support and so-called emotional AI. Part V concludes.

### Humans and Human-Like AI

The use of AI in situations that traditionally involve humans demonstrates the technology’s growing sophistication and adaptivity. There are also concerns about the risks this increased use of AI carries. The response to these risks may invoke policy or new social conventions. Such is the level of concern about some of the risks that law is also being deployed as a response. Thus, in particular, the EU AI Act^1^ imposes obligations on those developing, providing, and deploying AI models and systems to ensure they operate in a safe and responsible manner, with high levels of transparency about both the system and its oversight.

Further scrutiny of some kinds of human–AI interactions may be justified, even where the AI is able to provide accurate and fair responses to the humans with whom it interacts, although especially where there is doubt over these issues. The concern is that where AI is operating in fields that involve close human interaction, particular scrutiny may be required to ensure regard to basic precepts of respect for human dignity and autonomy.

Similar debates have taken place in the context of social robots. Here, philosophers and ethicists have asked what rules should govern human-robot interactions and whether there are kinds of interactions for which we should not use robots. For example, the use of social robots in aged care has been debated, on the one hand, as giving comfort to elderly residents and freeing caregivers to focus on high-value tasks, while on the other hand, as dehumanizing patients and disrespecting human dignity (e.g., Sharkey and Sharkey 2010; Yuan et al 2023). Relationships between humans and robots have been scrutinized from perspectives of the impact on humans (Danaher 2020) and on robots (Gunkel 2018). It is not surprising that these kinds of inquiries would extend to interactions with AI, which effectively involve disembodied robots (cf. Calo 2015, 529). Indeed, there is much to be learnt from debates about robot ethics when thinking about our relationships with AI (Sætra and Danaher 2022).

Deceit—deliberately misleading someone for gain—is a normative and legal wrong. It would be deceptive in this sense for an AI chatbot to provide incorrect information to a person for the purpose of benefiting the firm that has deployed the chatbot. In some instances, the law also sanctions misleading conduct that is not intentional or even negligent because of the harm it will cause to the person being misled and as an incentive to the firm in question to take more care.

It is relatively uncontroversial to suggest that AI should not deceive humans about its identity. The law in many judications already prohibits deceit and misrepresentation, including involving AI (Paterson 2023). AI mimicking humans, therefore, may contravene these prohibitions in circumstances where the human audience would either misunderstand or be unable to detect its status. The negative prohibition on deception or misrepresentation will often effectively require a positive obligation of transparency. Thus, those deploying AI should disclose its artificial status in circumstances where that may not be clear. The prime example of AI-specific law, the EU AI Act, requires disclosure of the artificial status of chatbots (Article 50(1)) and deepfakes (Article 50(4)).

The follow up question is whether it is deceptive, or otherwise wrongful, for an artificial entity to be presented as having the kinds of human traits relevant to interpersonal interactions, such as the capacity to feel or understand human emotion. And relatedly whether deployers should, at a minimum, disclose the artificial nature of these behaviours in circumstances where an AI is interacting with humans, at least in a personal or intimate way.

Transparency, or disclosure, about the artificial status of an AI has been treated as a sufficient response in law at least to concerns about humans being deceived. However, it might be asked whether, having regard to our current state of understanding about AI, AI should ever present as having the capacity to feel or to understand human emotions, even if the limitations on that capacity are revealed. This position might be taken on the ground that people interacting with an AI may never really abandon the idea it is authentic or real, disregarding disclaimers or warnings to privilege their emotional intuitions. Famously, Weizenbaum, who developed ELIZA, the early chatbot, observed that people attributed human-like feelings to ELIZA even though they were told it was merely a computer programme (Weizenbaum 1966, 42). If, effectively, we cannot prevent people from being misled by humanistic AI, perhaps there should be very compelling reasons for deploying such technology in sensitive interactions with humans.

There is an additional objection that, regardless of whether humans are misled, using AI in quintessentially human interactions erodes human dignity. This is on the ground that an AI that expresses emotions, feelings, and empathy is merely mimicking those traits, and that act of mimicking in some way denies the essential humanity of those interactions (Tarnoff 2023) by reducing them to a set of statistical prompts and responses. This concern has most force in circumstances where humans don’t understand the limitations of dealing with an AI. It also arises where humans have little or no choice about the interaction.

Typically, the law does not impose bans merely in response to normative as opposed to physical or economic harm, the sale of organs being an example. This response is commonly seen as unduly paternalistic. The objection to paternalism has less force where we are concerned about a lack of understanding or choice, which as we will see is a real consideration with AI applications currently. In any event, we can only assess the case for new laws or policies by working through these kinds of ethical uncertainties about the deployment of AI in what were previously exclusively human-to-human interactions. It is, therefore, helpful to consider a number of the different scenarios where AI may be used in this manner.

### AI Mimicking Humans

#### AI Influencers

The possibility of AI mimicking humans was once the material of science fiction. Now, however, AI uses are coming closer to this possibility. One example is the growth of virtual human-like AI influencers (Zahn 2024). For example, online influencer Lil Miquela has over three million followers on Instagram alone, while Lu do Magalu has over fourteen million (Molenaar 2024).

There is of course a human behind AI influencers. However, as is the case in most of these examples, the key relationship is between the AI and the humans with which it interacts, in this case the AI influencer’s online followers. These entities promote themselves as presenting new kinds of virtual creativity as they model fashion, lifestyle, art, and sexuality. Their purpose is typically to promote and sell products (Nguyen 2023).

Ethically, and by law, it will be essential to disclose the AI influencer’s virtual identity. Indeed, any influencers promoting items for a sponsor should disclose that sponsorship to avoid contravening prohibitions on misleading conduct in general law and consumer protection statutes (Australian Competition and Consumer Commission 2023, 3). This is because the sponsorship creates a conflict of interest that may have influenced, or may be perceived as influencing, their assessment of the product, and is relevant to followers'understanding of the integrity of the promotion (Australian Competition and Consumer Commission 2023, 3–4).

Similar reasoning would suggest that those using AI influencers to promote their products should disclose the influencer’s virtual status. The most extreme example of this concern is scams, which increasingly occur through AI avatars designed to resemble real people (Paterson 2024). However, even a transaction that is not a scam, in the sense that the consumer receives a genuine product, may still be problematic in a commercial and moral sense if informed by or induced by a false impression, in this case, that an actual human favoured the product being sold. The artificial status of an AI influencer may be a relevant factor influencing consumers’ reliance on its product promotion. The product may not be as good as presented if used by a human, not an AI. Alternatively, perhaps the consumer only wanted the product because of its association with a specific “human” influencer.

Arguably, AI influencers should disclose their artificial status even when they are not trying to sell a particular product but merely promoting a lifestyle or fashion trend. Principles of ethical robotics developed by scientists and scholars suggest that a modern version of the laws of robotics should require that robots do not mislead humans about their artificial identity or capacity (Pasquale 2020; Boden et al. 2017). It is consistent to suggest the same principle should apply to virtual “humans.” Without such an obligation, humans may be confused about the identity of the entity by whom they are influenced.

A response to these suggestions for transparency from AI influencers might be that no actual harm comes from human interactions with these entities regardless of how they are understood. However, this position underplays the wrong that occurs in the very act of misleading humans. Mispresenting the true circumstances, in this case that an actual human is living the life portrayed online, disrespects the dignity of the human interacting with the AI influencer. Such conduct treats the human as a mere means to another person’s ends, in this case the person who benefits from the influence of the AI (see also Hadfield et al. 1998).

The nondisclosed use of AI influencers also harms the people it replaces. Models, artists, and writers are increasingly having their jobs, and in some cases, their creative outputs, replaced by AI. AI influencers sometimes directly appropriate the images and designs of real models and celebrities (ABC News 2024)*.* In some cases, this will infringe intellectual property laws or prohibitions on misleading conduct (Paterson et al. 2018). Moreover, the increasing use of generative AI allows creators to generate content without directly copying, albeit being potentially inspired by real human work. As yet, both law and society are struggling with how to respond to the expropriation of human artists’ work for training generative AI (Handley 2024; Hacohen et al. 2024). However, the minimum requirement must be disclosure. Failing to reveal the artificial status of the work or the image denies viewers a critical opportunity to understand that work as an output of a technology, not the creativity of a human. It further denies the respect that society may feel should be accorded to genuine as opposed to virtual human creators.

The social media context may make pondering about the transparency of AI influencers seem trivial because, ultimately, influencers merely influence, not dictate, human decisions. Equally, there is no good reason why humans should be misled even in their online interactions. Moreover, these manifestations of virtual humans are merely the tip of the iceberg in terms of AI mimicking human interactions.

#### AI Companions

One of the most startling and unsettling developments in AI products on the market in recent times is the rise in so-called AI companions. The most prominent example of this trend is Replika. For a fee, Replika offers an AI avatar designed and maintained by the human user (Replika, n.d.). The consumer controls the appearance and broad parameters of the personality and interests of their AI companion. Once created, the Replika interacts seemingly naturally with the consumer by providing online text based chats and visual representations of a virtual life (Delouya 2023).

Attempts by an AI companion to influence or pressure human consumers to act against their interests and perhaps benefit the company that provides the AI would be contrary to consumer protection doctrines that protect against undue influence, undue pressure, or duress (Paterson and Maker 2024, 132; Paterson and Bant 2020, 18). Beyond these kinds of protection against interferences with free and informed consent, provided the artificial status of the companion is fully disclosed in the signup process and it performs as promoted, the law currently has little to say about artificial companions such as Replika. However, there are a host of as yet nascent concerns.

Replika has undergone several iterations to respond to concerns about sexual and emotional violence. These changes cannot be questioned, but frequent updates have also resulted in the unexplained loss of the pre-existing personality of some AI companions, which has caused distress and disappointment in users (Verma 2023; Brooks 2023). It would seem the boundary between companionship and a commercial product which may be varied at the will of the supplier is not well understood by at least some users.

The very premise of AI companionship has further raised concerns among scholars and ethicists (Alegre 2024; Chen et al. 2023; Ciriello et al. 2024; Paterson and Maker 2024). One of these concerns is that the human is somehow seduced into believing its relationship with the avatar is real, not artificial. Replika is promoted as a “companion” “who cares” (Verma 2023; Kaplan 2024). Dr Jodi Halpern observes there is no “care” in the interaction with an AI, particularly one that is a commercial product (Kaplan 2024). Replika is a product that reflects the preferences of the person who created it and the design of the company that put it on the market. It may be that consumers will, at some subliminal level, misunderstand the nature of the relationship with the avatar they have created. Joanna J. Bryson observes the tendency of humans to ascribe human-like qualities to robots (Bryson 2010, 63–74). They may also do this with AI avatars that purport to offer companionship.

A human who is led into believing the avatar is somehow real or that the relationship with that avatar is anything more than commercial is being deceived. But what about the impression that the AI companion “cares” or has feelings for the humans they serve? Danaher argues that there is no significant element of deception in a robot presenting as having feelings in its interactions with humans (Danaher 2020). The argument is that humans are no more misled by this conduct that in any other human to human interaction. Humans never truly feel or understand another person’s emotions. We merely respond to a manifestation of those emotions. So it is with robots, and by association, AI.

However, this kind of argument assumes humans understand that they are, firstly, dealing with an AI, and secondly, that an AI doesn’t (at least in this point of time) feel anything—it is merely simulating feeling. Humans by contrast may disguise or misrepresent their “true” emotions—but they share a common feature of experiencing interpersonal interactions as a human. We, or most of us, do not understand how AI interprets emotion or manifests feelings. It is here that people might be misled into thinking they are interacting with an entity that has a capacity to feel or understand human emotion in a human-like way, when they are not. This understanding may change over time, in which case the kinds of human–AI interactions that fall within prohibitions on deceptive conduct or requiring transparency will also change.

Additionally, these fake relationships raise the concern that the human is missing out on an opportunity for a real relationship. Some might suggest that this assumption about the value of human-to-human bonding is culturally determined and increasingly outdated (Jecker et al. 2024). A virtual relationship may not fully address the human needs for companionship and care (Paterson and Maker 2024, 133). For those turning to Replika for companionship, this artificial relationship may escalate rather than reduce loneliness, which Ciriello et al. describe as the “companionship-alienation” irony (Ciriello et al. 2024, 2).

A related concern is that humans become dehumanized by being immersed in artificial relationships. Chen et al. explain that if we “place [Artificial Emotional Intelligence] on equal footing with genuine human empathy and consciousness, we might contribute to a dilution of the authenticity and complexity of these essential human emotions and traits” (Chen et al. 2023, 2; Alegre 2024).

### AI and the Capacity to Interpret Humans

So far, the conversation has focused on AI interacting with humans in a way that mimics human characteristics. AI is also being presented as having the capacity to interpret aspects of human behaviour, including emotions.

#### AI Therapists

In recent years, apps have been proliferating to support users’ mental health through automated processes and AI (Auxier et al. 2021). Some apps provide curated meditations (e.g., Calm n.d.) or mindfulness practices (e.g., Headspace n.d.). Others provide counselling or therapy through an interactive bot (e.g., Youper n.d.; Wysa n.d.; Woebot n.d.).These services are commonly entirely automated and provided without the direct involvement of a health professional. While initially deploying preprogrammed expert systems (Darcy 2023), these apps are increasingly likely to use generative AI (Blease and Torous 2023; cf. Shamayleh 2023). This use aims to provide consumers with more personalized and responsive support for good mental health.

Mental health apps may appear to be an undeniably beneficial application of new digital technologies. Many people struggle to access mental health support (Campbell 2022; Hickie 2023; Davey 2023; Abrams 2022). Mental health apps respond to this shortage by providing personalized support based on established therapeutic practices. Therapeutic goods regulators commonly class such products as low risk and, therefore, subject to only minimal regulation (see, e.g., Medicines and Healthcare Products Regulatory Agency 2023 (U.K.); Therapeutic Goods Administration 2022 (Australia); and, in the United States, Shuren, Patel, and Gottlieb 2018; Mattioli 2021), although the United Kingdom has recently issued revised regulatory guidance for the oversight of digital mental health technologies (Medicines and Healthcare Products Regulatory Agency 2025).

There are some powerfully persuasive reasons why such apps should be subject to greater legal and ethical scrutiny (Paterson and Ananthapadmanaban 2023). The stakes are high in terms of error or harm. They also raise the question of when and how AI should be deployed in fields traditionally relying on trusting therapeutic and, ultimately, human relationships very starkly.

The potential for harm to individuals arising from mental health apps is significant on several fronts (Paterson et al. 2023). The products’ clinical efficacy is still to be established (Goldberg et al. 2022; Mattioli 2021). The apps offer personalized mental health strategies to a diverse cohort of potential users with little formal oversight of their operation. Moreover, the apps target consumers who may be experiencing vulnerability by any measure. Mental health apps are aimed at people concerned about their mental health and who may, during their use of the app, experience a mental health crisis (Cox 2024). Studies have found that privacy policies of mental health apps commonly allow large amounts of personal and sensitive data to be collected and shared (Steindl 2023; Caltrinder et al. 2023).

Many providers assert an idealistic ambition to improve mental health through more personalized and low-cost support (Paterson and Ananthapadmanaban 2023, 3). If this is genuinely the case, then the service should not be provided in a way that disregards the risks inherent in the technology. One way for mental health app providers to virtue signal would be to commit to best ethical practice in the clinical aspects of mental health support and in providing that service as a consumer product through technological means. This means providing a reliable, effective service and being respectful of individual privacy.However, even if this is done, there remains a question about whether mental health support *should* be provided through an AI chatbot app.

The question matters. The rise of mental health apps may have the effect of diverting resources away from skilled human providers of mental health support. The increasing availability of mental health apps may shape the market for mental health support services, pushing the norm towards automation rather than more support for human providers of support services. Pasquale observes that the ethical issues raised by mental health apps include issues of equity, such as “whether a linguistic corpus of stimuli and responses adequately covers diverse communities with distinct accents and modes of self-presentation” (Pasquale 2019). They also raise legal and political critiques involving “whether the apps are prematurely disrupting markets for (and the profession of) mental health care in order to accelerate the substitution of cheap (if limited) software for more expensive, expert, and empathetic professionals” (Pasquale 2019). It is, therefore, critical that the apps work in genuinely beneficial and ethical ways for consumers seeking mental health support and that they are beneficent; that is, they improve the position of humans rather than disappoint or deceive them.

Thus, a key concern with mental health apps relates to the relationship between the AI chatbot and consumers. As with AI companions and influencers, there is a concern that consumers may be mistaken about the capacity of the AI, thinking it is human or human-like. However, there are also problems in the reverse situation, whereby consumers know that the AI mental health chatbot is an artificial agent but attribute superhuman powers of insight or intuition to it for the very reason that it is an “AI.” Wysa promotes its chatbot as using “a combination of rule based algorithms and large language modelling to *listen and respond intelligently* to the thoughts and emotions that you express” (Wysa FAQ n.d.) (emphasis added).

This misapprehension may lead to excessive levels of trust and confidence being placed in the capacity of the chatbot, which may, in turn, impede the efficacy of the mental health support being provided.

A more complex ethical question is whether we in some way degrade our humanity by relegating our minds—part of the very foundations of being human—to inquiry by an artificial entity. Therapy apps using chatbots make claims to the supportive character of the relationship. For example, Woebot’s website links to preliminary clinical evidence showing a therapeutic bond between the chatbot and users (Darcy et al. 2021). Tekin notes that “therapeutic alliance is a strong predictor of successful outcomes … Building a therapeutic alliance is a relational process, in which the therapist gives uptake to the patient’s concerns, and the patient feels recognized and cared for” (Tekin 2021, 456). Tekin is however sceptical that “this type of alliance can be formed between a person and a bot” (Tekin 2021, 456). The very aspiration raises questions about whether it is ethical or even productive, in a therapeutic sense, to create expectations of an interpersonal relationship with what is, in reality, an artificial human.

I have already suggested that it may be wrong to present an AI as having human-like capacity for insight and understanding (see Part II). AI systems are not humans or even replica humans. Our relationship with technology is not the same as with other humans. Even if humans are not tricked into thinking the AI is human, there may be something troubling about promoting the skill of an AI therapist in assisting people at a time when they are concerned about their mental health. This might suggest that chatbots are not the answer, even if they provide some therapeutic benefit, which, in any event, needs to be established. The core issue is that chatbots do not know us like humans would. Or do they? There is ongoing interest in some AI developers using AI to identify human emotions.

#### Emotion Detection AI

The field of “emotional AI” seeks to use AI to identify human emotion, character, or illness from biometric data, such as from faces, eye movements, voice, gait, or blood pressure (Schuller and Schuller 2018; Somers 2019). This kind of application of AI goes further than the use of natural language processing techniques in allowing AI to respond to human language used in chatbots for mental health. It also goes beyond the use of computer vision, allowing humans to be identified by their faces or other biometric characteristics (Davis et al. 2022). Instead, human biometric information is used to determine the emotional state of the human subject (Emerging Technology from the airXiv 2015, Stanley 2019, 21–25). Such techniques involve the use of personal data, for consent must usually be sought under legal data protection regimes. But this only addresses some of the privacy and ethical issues raised by AI for emotion detection (Chang 2022, 49; Hauselmann et al. 2023). There is an ongoing debate about what regulatory mechanisms should be in place to protect individuals from this poorly understood technology (Clifford 2024; Podoletz 2022).

Emotion detection software may rely on several markers. Eye tracking, for example, is used to detect sleepiness in truck drivers (Vetturi et al. 2020). Blood pressure and heart rate may indicate an aroused state. The critical controversy lies in the use of facial analysis to detect emotion. This application of emotional AI has been variously critiqued as “junk science” (Santow 2020, 17), “pseudoscience” (Whittaker et al. 2018, 14), and as relying on “naïve technocratic simplification based on dubious beliefs about emotions” (Stanley 2019, 38). AI used for detecting emotion from faces commonly relies on discredited beliefs about the salience of “micro-expression” (Whittaker et al. 2018, 14), grounded on culturally specific and stereotypical beliefs about human behaviour (Jack et al. 2012; Heaven 2020; Siegel et al. 2018).

A study into the correlations between emotion and facial expression by Barrett et al. concluded that “the facial configurations [commonly used to represent emotions] are best thought of as Western gestures, symbols or stereotypes that fail to capture the wide variety with which people spontaneously move their faces to express emotions in everyday life” (Barrett et al. 2019, 46). In this context the use of emotion detection perpetuates and amplifies these stereotypes. This, in turn, leads to risks of discrimination against people misidentified by even benevolently intended uses of the technology (Whittaker et al. 2018, 14–15). Sensitive to these issues, in 2022, Microsoft decided to retire facial analysis software that purported to infer emotional states (Bird 2022). Microsoft considered that this technology raised “important questions about privacy, the lack of consensus on a definition of ‘emotions,’ and the inability to generalise the linkage between facial expression and emotional state across use cases, regions, and demographics” (Bird 2022, ¶6 under “Safeguards for responsible use”).

Article 5(1)(f) of the EU AI Act bans the use of emotion detection systems in workplaces or schools. The ban responds to concerns about humans being ranked, rewarded, or punished for their perceived emotional engagement or characteristics in the context of critical and, in principle, inclusive public institutions (Whittaker et al. 2018). Article 50(3) of the EU AI Act imposes transparency requirements on those who use emotion detection systems (see also Hauselmann et al. 2023). Transparency requirements are essential as a way of allowing individuals and advocacy groups to identify when and how emotion detection systems are being used in ways that affect civil liberties and human rights. This is important in emotion detection, where inaccuracy and bias may be rife.

Even if these failings were addressed, the ethics of emotion detection require ongoing attention (see, e.g., Clifford 2024). Considerable attention is being given to the future of neurotech, which involves wearables or implants that can influence one’s state of mind (Office of the United Nations High Commissioner for Human Rights n.d.; Australian Human Rights Commission 2024). Emotion detection is not far removed. Even the potential use of AI raises concerns as the ability to recognize emotion would provide considerable leverage over human behaviour and concepts of humanness.

One concern is the possibility of emotion-detecting bots manipulating humans for fraud, disinformation, or commercial advantage (Hauselmann et al. 2023). We have already seen that deliberately manipulating consumer preferences by taking advantage of a vulnerable state is generally unlawful. Using emotion detection software to target consumers with advertisements designed to influence their current emotional state would likely be regarded as going beyond mere strategic marketing to offend this rule (see also EU AI Act, article 5). Nevertheless, as Valcke et al. note, traditional approaches to consumer protection may struggle to provide “a conclusive and exhaustive answer to the question of where to draw the line between forms of permissible persuasion and unacceptable manipulation in the case of emotional AI” (Valcke et al. 2021, 62).

The technology might be defended as having some plausible uses. For example, Davis, Perry, and Santow refer to the potentially justified use of facial recognition technology (which might include emotion AI) in policing and security work (Davis et al. 2022, 71). Clifford refers to possible uses in healthcare and road safety (Clifford 2020, 83). The ability to identify emotion might allow the more successful deployment of AI for companionship and mental health support, amongst other uses.Here, it might be argued that some concerns about these kinds of uses dissipate if AI is attuned to emotion. The capacity to detect and respond to human emotion may make the AI a more supportive human companion as it responds to changes in mood or feeling.An emotion-detecting mental health chatbot might provide more personalized and practical advice. Emotion detection means the bot may also be able to more effectively pre-empt more harmful occurrences, such as anxiety, stress, depression, or psychosis.

Even in the face of these possibilities, whether humans should be striving to develop, deploy, or use emotion-detecting bots may still be questioned. Pasquale describes an ethical problem of AI “counterfeiting” distinctively human characteristics (Pasquale 2020, 7–9). People dealing with a chatbot that responds to their emotions may ascribe a false level of consciousness or empathy to that entity. Such people then may have a counterfeited relationship. People may wrongly assume the AI understands or cares about them, or they may overidentify with the AI (Bryson 2010), when, in fact, there is no ability to care at all. We can be reminded of the early chatbot therapist ELIZA, to which individuals attributed human-like feelings even though they were informed it was a computer programme (Chesterman 2021, 114). In this world, we may object to the misrepresentation of care. More chillingly, it might be argued that such developments reduce us to the subjects of a sweeping technological management system, under which previously human-centred interactions are determined by technologically based categorization (Brownsword 2019, 23; Dagan 2019, 1297–1298). Goldenfein describes the use of AI classification as supporting “computational empiricism or positivism as a dominant knowledge system likely to have ongoing effects in juridical and political practices” (Goldenfein 2019, 111).

## Conclusion

AI, in the form of machine learning algorithms for predicting behaviour or large language models generating new outputs, is increasingly being deployed to interact with humans. These interactions often involve AI mimicking human characteristics, such as appearance, empathy, and insight, to provide entertainment, companionship, and support. Examples considered in this paper have included AI social media influencers, AI companions, and AI chatbots for mental health support. Going further, AI has sometimes been proposed to identify and interpret human emotions. Whether considered spectacular or unsettling, these developments require greater legal and ethical scrutiny. Legal and regulatory frameworks can respond to AI that deceives us by presenting as human or as having human like capacity. There are also ethical questions that go beyond these legal preoccupations with misrepresentation and transparency. Even when correctly labelled as artificial, should we embrace AI that mimics human appearance, simulates feelings, or seeks to interpret human mental states or emotion? Humans may want to consider whether some of the currently proposed interactions with the technology represent the future they want. There is still time to influence the nature of our relationship with AI.

## Data Availability

Not applicable

## Footnotes

1 P9 TA(2024)0138. European Parliament legislative resolution of 13 March 2024 on the proposal for a regulation of the European Parliament and of the Council on laying down harmonised rules on Artificial Intelligence (Artificial Intelligence Act) and amending certain Union Legislative Acts. https://www.europarl.europa.eu/doceo/document/TA-9-2024-0138_EN.pdf
